# Cationic Micelle-like Nanoparticles as the Carrier of Methotrexate for Glioblastoma Treatment

**DOI:** 10.3390/molecules29245977

**Published:** 2024-12-18

**Authors:** Tuğba Nur Aslan

**Affiliations:** 1 Department of Molecular Biology and Genetics, Faculty of Arts and Science, Necmettin Erbakan University, Konya 42090, Turkey; taslan@erbakan.edu.tr; 2 Science and Technology Research and Application Center (BITAM), Necmettin Erbakan University, Konya 42140, Turkey

**Keywords:** CTAB, glioblastoma, iron oxide nanoparticles, methotrexate

## Abstract

In the present study, ultra-small, magnetic, oleyl amine-coated Fe_3_O_4_ nanoparticles were synthesized and stabilized with a cationic ligand, cetyltrimethylammonium bromide, and an anticancer drug, methotrexate, was incorporated into a micelle-like nanoparticle structure for glioblastoma treatment. Nanoparticles were further characterized for their physicochemical properties using spectroscopic methods. Drug incorporation efficiency, drug loading, and drug release profile of the nanoparticles were investigated. According to the results, max incorporation efficiency% of 89.5 was found for 25 µg/mL of methotrexate-loaded nanoparticles. The cumulative amount of methotrexate released reached 40% at physiological pH and 85% at a pH of 5.0 up to 12 h. The toxicity and anticancer efficacy of the nanoparticles were also studied on U87 cancer and L929 cells. IC_50_ concentration of nanoparticles reduced cell viability to 49% in U87 and 72% in L929 cells. The cellular uptake of nanoparticles was found to be 1.92-fold higher in U87 than in L929 cells. The total apoptosis% in U87 cells was estimated to be ~10-fold higher than what was observed in the L929 cells. Nanoparticles also inhibited the cell motility and prevented the metastasis of U87 cell lines. Overall, designed nanoparticles are a promising controlled delivery system for methotrexate to the cancer cells to achieve better therapeutic outcomes.

## 1. Introduction

Glioblastoma multiforme (GBM) is the most common malignant primary brain tumor in adults [1]. Some treatment methods, such as resection, chemotherapy, radiotherapy, and their combinations, are applied for their treatment, and they have been able to extend survival by only 14 months [2,3]. Its treatment is often difficult due to biological barriers such as the blood–brain barrier (BBB) and tumor cell membranes [4,5]. Poor bioavailability and accumulation of drugs in the tumor site due to their nontarget-specific nature, development of drug resistance, and toxicity to healthy cells are the main obstacles for GBM treatments [6,7]. Thus, effective treatment strategies are sought for GBM, such as nanotechnologic approaches utilizing a variety of nanoparticles, such as magnetic nanoparticles [8,9]. Magnetic nanoparticles stand out as a promising technology in targeted drug delivery systems for brain tumors [10,11]. Magnetic nanoparticles as carriers for drug molecules can be directed and retained at the tumor site using an external magnetic field [12]. This minimizes damage to healthy cells and reduces side effects [13]. Magnetic nanoparticles can also be used as magnetic resonance imaging (MRI) agents to monitor the movements and distribution of nanoparticles within the brain in real time [14]. Among magnetic nanoparticles, due to their low toxicity, biodegradability, and anticancer, antibacterial, and cell labeling properties, iron oxide nanoparticles, which exhibit both magnetic behavior and semiconductor properties, have found widespread application in multifunctional biomedical fields [15]. Magnetite (Fe_3_O_4_) nanoparticles received the most attention among the various forms of iron oxide (goethite, wustite, maghemite (γ-Fe_2_O_3_), hematite (∝-Fe_2_O_3_) because of their high saturation magnetization (Ms) value, injectability, chemical stability, interaction with biomolecules, and ease of synthesis and targeted drug delivery [16,17]. Fe_3_O_4_ nanoparticles larger than 50 nm can be employed primarily for in vitro magnetic separation due to their ferromagnetic properties. However, superparamagnetic Fe_3_O_4_ nanoparticles require particle sizes smaller than 50 nm, which is the diameter of the reticuloendothelial system (RES) in the brain that can be utilized in in vivo biomedical applications for neuroresearch [18]. Moreover, superparamagnetic Fe_3_O_4_ nanoparticles find applications in magnetic hyperthermia therapy by exhibiting the highest heating rates [16,19]. Recently, Fe_3_O_4_ nanoparticles were found to induce a cytotoxic effect through a unique form of controlled necrosis called ferroptosis, which results from high levels of iron ions and ROS. This iron-programmed cell death leads to iron-dependent lipid peroxidation (LPO) that demonstrated promise in preclinical investigations for treating therapeutically resistant cancer cells with exceptional treatment outcomes [20,21]. Additionally, the combination of drug incorporation into Fe_3_O_4_ nanoparticles and applying a magnetic field allows drug molecules to be released in a controlled manner at the target tissue, making the treatment process more effective and safer by reducing treatment-related side effects [22]. Moreover, a variety of drugs have been used for brain tumors [23]. Among them, MTX is selected as an analog of folic acid that can target folate receptors overexpressed on different types of cancers [24,25]. This drug has been utilized as an antitumor agent for the treatment of some cancer types such as breast, skin, brain, lung, ovary, and leukemias [24,25,26]. However, there remain some limitations about its usage, including its side effects, poor water solubility, short half-life in the bloodstream, and subsequently poor bioavailability [24,25,27]. In addition to these, free MTX may enter the cytosol and inhibit the dihydrofolate reductase enzyme (DHFR) and folic acid cycle, which reduces cellular viability and leads to cell death [24,25]. Therefore, magnetic nanoparticles can be suggested for the development of an efficient nanocarrier for the delivery of MTX [24,27].

Some kind of encapsulation and incorporation methods using several coating materials and ligands have been studied for safe targeting while resolving the above-mentioned challenges [27]. Among the nanocarrier systems, micelle-like structures had been used for improving the bioavailability of MTX, reducing the rapid diffusion throughout the body while delivering the effective concentration to the tumor region [28,29]. Charged nano-micellar structures with smaller sizes (<30 nm) are also advantageous in exhibiting excellent stability against aggregation and targeted accumulation in tumor sites by enhanced the permeability retention (EPR) effect, reducing the dose of the drug administered to the body and hence reducing the toxicity of the drug itself when compared to other traditional chemotherapy [30,31,32]. Cationic nanoparticles possess strong cellular interaction and cell membrane disruption properties, providing good cellular uptake, thus making the nanoparticle selective for the cell membrane when compared with neutral or negatively charged particles; its mechanism is attributed to adsorption-mediated endocytosis [4,33,34,35]. In the literature, some studies with cationic nanostructures, such as cationic bovine serum albumin, superparamagnetic iron oxide NPs conjugated with cationic lactoferrin, TAT-modified cationic peptide, cationic albumin-conjugated pegylated nanoparticles, doxorubicin-loaded cationic mannose-modified albumins, and cationic polymeric magnetic liposomes, indicated their transportation across the BBB [36,37,38,39].

In the present work, a simple and interfacial ligand modification approach has been presented to prepare a highly monodisperse, cationic magnetic iron oxide, Fe_3_O_4_, nanocarrier for the delivery of MTX to the tumor location with an external magnetic field to serve as a passive targeting agent with an enhanced permeability retention (EPR) property due to its smaller size for glioblastoma, whereas the cationic charge of nanoparticles will also help in active targeting [29,40]. MTX was incorporated by both physical entrapment and electrostatic adsorption to the nanoparticle structure at the same time in one drug delivery system. Oleylamine (OAm) functionalized iron oxide nanoparticles were first synthesized by the thermal decomposition method. In the incorporation approach, the oleylamine chains were functionalized with CTAB ligand, resulting in a “bilayer” structure and retaining the small core size of the nanoparticles [41]. A hydrophobic packet through the interfacial area of the ligands was formed that will accommodate MTX by physical entrapment and/or chemical hydrophobic interactions at the same time [41]. In addition, phase transfer of nanoparticles into the aqueous phase had been achieved through the attached CTAB ligand with hydrophilic positively charged tails on the nanoparticle surface while providing the electrostatic interaction with the drug since MTX is a negatively charged molecule at physiological pH [42]. Shortly, the ligand modification and phase transfer, along with MTX incorporation, were achieved in a single step as a simple and effective method, and the approach yields water-soluble and stable nanoparticles. The designed nanocarrier system provided an advantage by enabling a quick release from surface-adsorbed drug molecules to reach effective drug plasma concentration and sustained release from the entrapped drug molecules to reach effective drug concentration; otherwise, MTX normally has a rapid migration property that results in the loss of drug in the aqueous phase [28,43]. Moreover, the positively charged nanocarrier system was also developed to lower the excess accumulation of MTX in normal cells and reduce the toxicity of the nanocarrier, while achieving effective inhibition of cancer cells. The results indicated that the synthesized magnetic nanocarrier improved the anticancer efficacy of MTX on U87 cell lines while hindering the toxicity, apoptosis, and drug uptake in L929 cell lines according to the cytotoxicity, Annexin-V, and cellular uptake experiments. Lastly, the nanoparticles inhibited the cell motility and prevented the metastasis of U87 cell lines. Furthermore, although the in vitro outcomes of the prepared nanoparticles are encouraging, in vivo investigations are necessary to confirm the system’s safety and effectiveness in more complex biological environments in further studies.

## 2. Results and Discussion

### 2.1. Characterization of Oleylamine Functionalized Fe_3_O_4_ Nanoparticles

**Figure 1:**
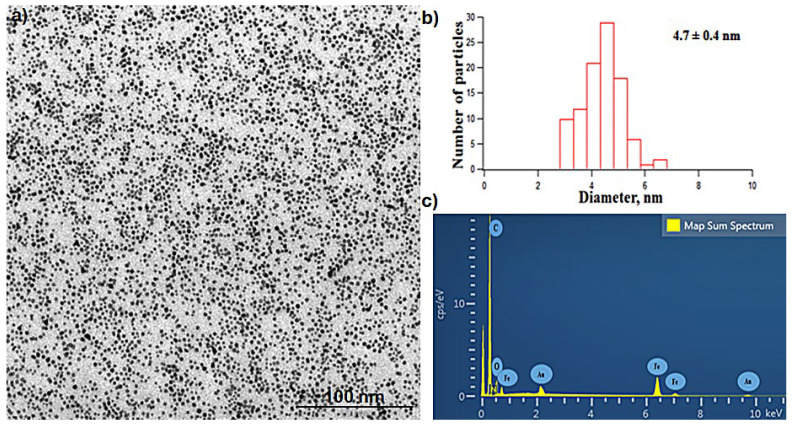
(**a**) Transmission electron microscope image (TEM); scale: 100 nm. (**b**) Size distribution diagram obtained from TEM image; (**c**) Energy dispersive spectrum (EDS) of OAm-Fe_3_O_4_ nanoparticles.

OAm-functionalized Fe_3_O_4_ nanoparticles with a homogeneous particle size distribution could be synthesized by the thermal decomposition method. The obtained nanoparticles with a hydrophobic property were suspended in hexane. The TEM image displays the spherical-shaped nanoparticles with an average size of 4.7 ± 0.4 nm that is estimated from the size analysis of 100 particles (Figure 1a,b). In the EDS spectrum, iron and oxygen peaks are observed, accompanied by gold and carbon peaks resulting from gold-coated carbon band used for sample holding (Figure 1c).

**Figure 2:**
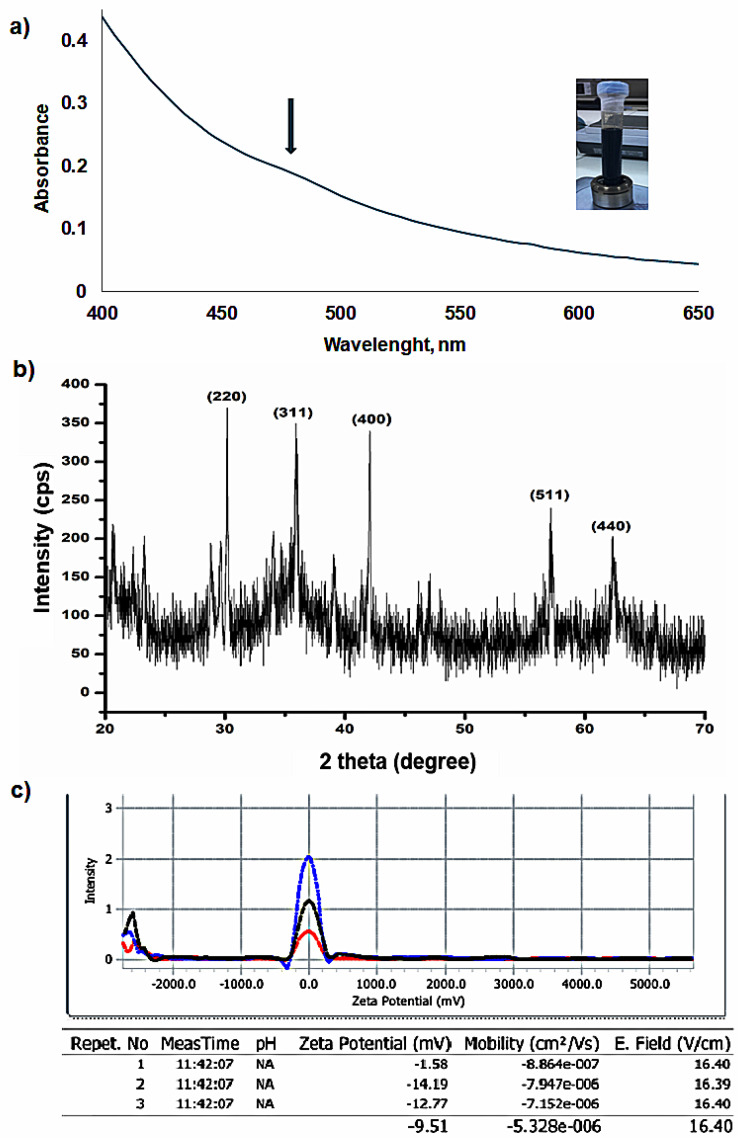
(**a**) The UV–Vis spectrum, the arrow indicates the maximum absorption band; (**b**) XRD pattern, and (**c**) Zeta potential of OAm-Fe_3_O_4_ nanoparticles dispersed in hexane.

Further characterizations were carried out by UV–Vis spectroscopy and XRD analysis. Figure 2a presents the UV–Vis spectrum of the OAm-Fe_3_O_4_ nanoparticles suspended in hexane. A typical spectrum obtained for OAm-Fe_3_O_4_ nanoparticles shows a broad absorption band between 400 and 500 nm [25,44]. The crystal structure of nanoparticles was elucidated by the XRD pattern of the nanoparticle powder sample (Figure 2b). The sharp peaks in the XRD spectrum indicate the crystallinity of the nanoparticles. The diffraction peaks with the 2θ values of 29.63°, 34.54°, 41.84°, 56.67° and 62.26° correspond to the (220), (311), (400), (511) and (440) planes, respectively according to the data in JCPDS card number 19-629 [45,46]. These obtained planes are accepted as the cubic and most magnetic phase of Fe_3_O_4_ [47].

The net surface charge of the nanoparticle surfaces indicates the electrostatically stabilized nanoparticles by interparticle repulsion. In other words, the surface charge has a major effect on the surface potential [48,49]. Surface charges also change with surface modification of nanoparticles with some charged molecules. To follow this change, the zeta potential of core nanoparticles in hexane was measured and found to be −9.51 mV (Figure 2c).

### 2.2. Phase-Transfer and MTX Incorporation

Ligand functionalization for the phase transfer process requires strong ligand–ligand interactions [50,51]. For instance, the trial experiment for the second oleylamine functionalization of OAm-Fe_3_O_4_ nanoparticles fails with a high agglomeration in the water phase. Interestingly, further functionalization with CTAB displayed an effective attachment to oleylamine, and CTAB-functionalized nanoparticles were well dispersed in the water phase without any agglomeration observed for months, as it is known that charged nanoparticles exhibit excellent stability against aggregation. Here, it was significant to optimize the sufficient concentration of CTAB and contact time of ligand and nanoparticle [52,53]. It has also been shown in the ‘Characterization of CTAB-MTX-OAm-Fe_3_O_4_ Nanoparticles’ section below that CTAB-MTX-Fe_3_O_4_ nanoparticles are thermally stable up to 250 °C, indicating the nanoparticle stability at room temperature.

Moreover, it is predicted that the strong interaction between oleylamine and CTAB may provide a strong MTX entrapment at the hydrophobic–hydrophobic interfacial area. MTX is an amphiphilic molecule that enables the hydrophobic interactions between the hydrophobic chain in the core, and it could also be physically adsorbed at the interface of oleylamine and CTAB [54,55,56]. The hydrophobic interactions of MTX have also been reported so much in the literature [42,56,57,58]. The incorporation of MTX does not only arise from the physical entrapment but also arises from the electrostatic interaction of MTX with the positively charged tails of CTAB since MTX is a negatively charged molecule at physiological pH due to its isoelectric point of ‘2.95’ [42,56,59].

The way of incorporating MTX into the nanoparticle surface was indirectly investigated by the analysis of the free MTX in supernatant using UV–Vis spectroscopy after a centrifugation process of nanoparticle suspension. Incorporated MTX was determined from the initial and free amount of MTX. In the entrapment procedure, the entrapment % is found to be 93 ± 2.3%, while the value is 32 ± 2.7% for the adsorption procedure from the absorbance values of MTX in supernatant at 303 nm. It can be concluded that adsorption of MTX already exists at around 32%; entrapment and adsorption occur together in entrapment procedure and thus the remaining attachment of 61% belongs to the entrapment process. The higher loading of MTX onto the nanoparticles could be reached since both the entrapment and adsorption of MTX occurs in one synthesis, such as similar approaches utilized in the literature. Therefore, CTAB-MTX-OAm-Fe_3_O_4_ nanoparticles were prepared according to the entrapment procedure and used for the subsequent experiments.

### 2.3. Incorporation Efficiency and Drug Loading Yield

CTAB-MTX-OAm-Fe_3_O_4_ nanoparticles were prepared by adding various amounts of MTX as follows; 10, 25, 50, 75, and 100 µL of MTX (1 mg/mL) to determine the incorporation efficiency and drug loading amount by HPLC method. Here, the initial concentrations of MTX added to the nanoparticle suspensions are 5 µg/mL, 12.5 µg/mL, 25 µg/mL, 37.5 µg/mL, and 50 µg/mL, respectively.

**Figure 3:**
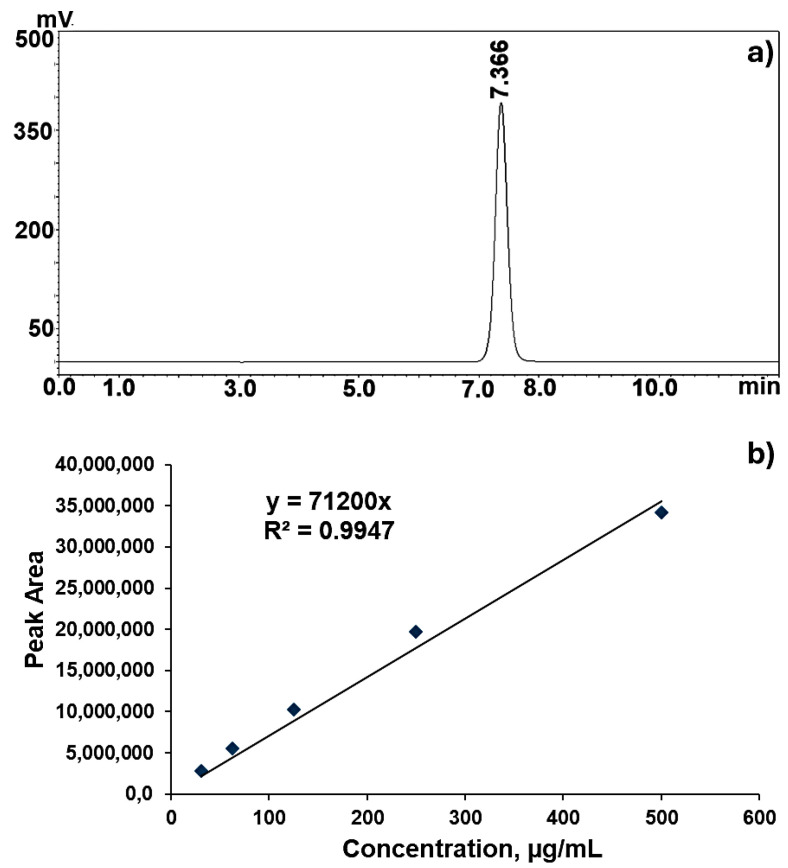
(**a**) HPLC chromatogram; (**b**) Calibration curve of methotrexate (MTX). The absorbance values were obtained at 305 nm.

In the method, the chromatograms of the standard solutions of MTX under optimized conditions were obtained, and the retention time of methotrexate was found to be 7.366 min as seen in Figure 3a. The HPLC calibration graph of MTX standards at concentrations of 31.25 µg/mL, 62.5 µg/mL, 125 µg/mL, 250 µg/mL, and 500 µg/mL is given in Figure 3b. Using the equation of the calibration graph obtained, the concentrations of the MTX incorporated into the nanoparticle were calculated.

**Figure 4:**
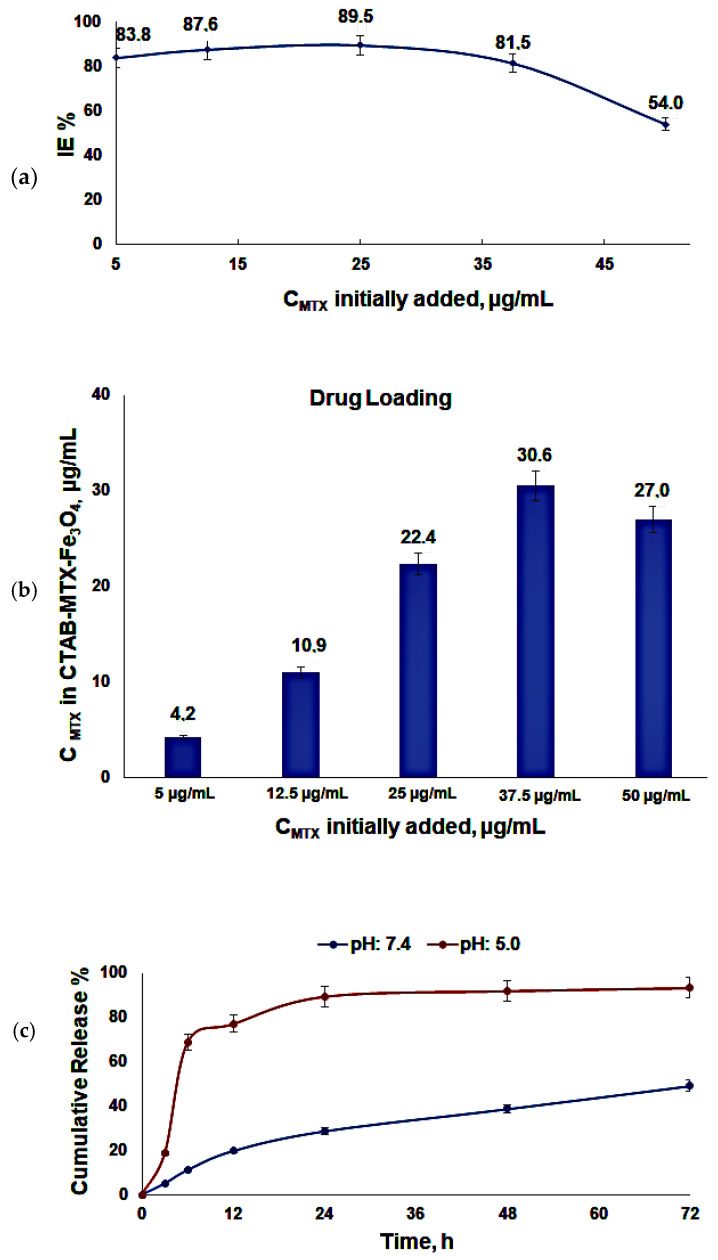
(**a**) Incorporation efficiency (IE) % of MTX in CTAB-MTX-OAm-Fe_4_ nanoparticles; (**b**) Incorporated drug concentrations (µg/mL) of CTAB-MTX-OAm-Fe_3_O_4_ nanoparticles versus initially added MTX concentrations; (**c**) Drug release profile of CTAB-MTX-OAm-Fe_3_O_4_ nanoparticles dialyzed against a pH of 7.4 and 5.0.

The graph of incorporation efficiency% of MTX versus initial concentration of MTX was obtained (Figure 4a). According to the graph, 89.5% of max IE% is found for 25 µg/mL of MTX loaded into nanoparticle suspension, while 81.5% of IE% is found for 37.5 µg/mL of MTX. Moreover, the max. amount of MTX incorporated into the nanoparticle was calculated as 30.6 µg/mL when 37.5 µg/mL of MTX is loaded into the nanoparticle micelle structure (Figure 4b). However, the binding capacity of MTX decreases to 27 µg/mL when 50 µg/mL of MTX is loaded into the structure (Figure 4b). The decrease in binding capacity at a concentration of 50 µg/µL of MTX could be attributed to saturation of the nanocarrier’s binding sites [60]. Any extra MTX may stay unbound after the nanocarrier’s accessible binding sites are completely occupied, leading to a plateau or decrease in measured binding capacity. Additionally, the nanocarrier may aggregate or undergo structural alterations because of high MTX concentrations, which could limit its capacity to bind other molecules [61]. Another possible explanation is competitive or steric hindrance effects at higher MTX concentrations, which could reduce binding efficiency [62]. Therefore, the nanoparticles were incubated with 37.5 µg/mL of MTX (75 µL, 1 mg/mL) solution for subsequent studies.

### 2.4. In Vitro Release

The release of MTX from CTAB-MTX-OAm-Fe_3_O_4_ nanoparticles at different time points, for up to 72 h, was evaluated by dialysis against PBS (pH 7.4) and acetate buffer (pH 5.0) at room temperature. Free MTX was collected from external buffer and analyzed by HPLC method. The results are summed as cumulative release % versus time (h) (Figure 4c). As seen in graphs, the release of MTX is higher in the acidic medium (pH: 5.0) than that of the physiological pH. This can reduce the side effects on normal cells since MTX will be readily released in an acidic environment. The release of drugs may show a biphasic profile that includes a quick/burst release in the first hours and a sustained release [61]. In comparison, the cumulative amount of initial release reached 22% in the first 6 h and 40% up to 12 h at physiological pH and 75% in the first 6 h and 85% up to 12 h at a pH of 5.0 with a significant amount of drug released within 12 h [63]. MTX through entrapment incorporation results in strong hydrophobic interaction and stable micelles; therefore, the profile displays a faster release up to 12 h since the release of adsorbed MTX (32 ± 2.7%) on the external surface of nanoparticles is taken first, and ongoing release after 12 h continues with low drug leakage up to 72 h due to the strong interaction at the shell/core interface [28,64]. After 12 h, the slower release may likely result from the hydrolysis of micelle formation until the structure breaks apart. Such a phenomenon was also observed in the literature [28]. Moreover, the initial faster release of adsorbed MTX may provide an effective drug plasma concentration, and an effective drug concentration may be maintained by the sustained release of entrapped MTX molecules [28].

### 2.5. Characterization of CTAB-MTX-OAm-Fe_3_O_4_ Nanoparticles

MTX could be incorporated in the cationic micelles through a ligand modification procedure and phase transfer of OAm-Fe_3_O_4_ nanoparticles by adding CTAB and MTX simultaneously. The optimization experiments for the ligand modification were carried out by varying some parameters, such as nanoparticle and CTAB concentration, stirring type (mixing or ultrasonic), and order of addition of reactants, while keeping all the other parameters constant except the one to be investigated.

**Figure 5:**
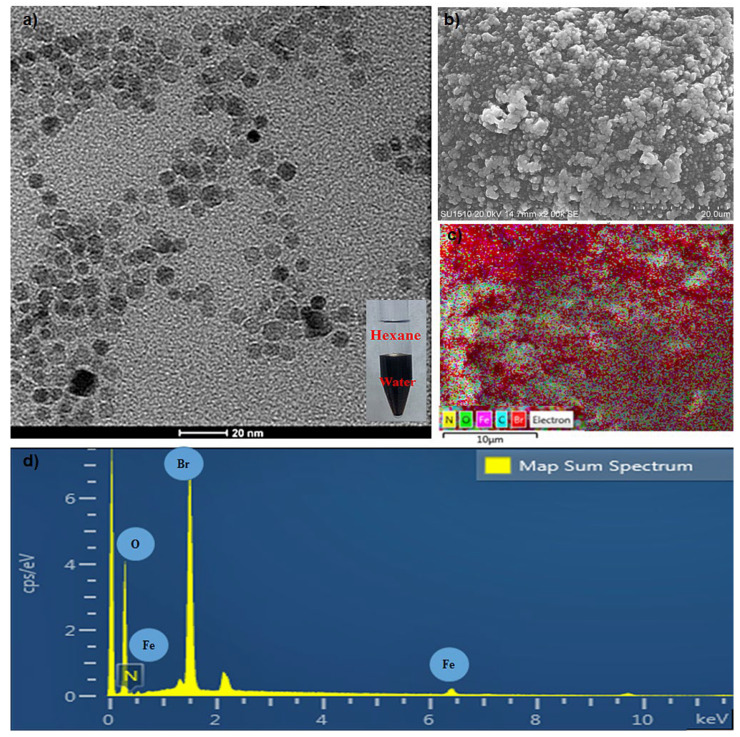
(**a**) Transmission electron microscope image (TEM); scale: 20 nm. (**b**) Scanning electron microscope image (SEM); scale: 20 µm. (**c**) Energy dispersive spectrum (EDS) analysis. (**d**) EDS spectrum of CTAB-MTX-OAm-Fe_3_O_4_ nanoparticles.

The TEM image of CTAB-MTX-OAm-Fe_3_O_4_ nanoparticles reveals their shape and size distribution, confirming the successful synthesis of monodispersed nanoparticles (Figure 5a). Furthermore, a TEM image taken approximately 10 days later showed that the nanoparticle suspension remained non-aggregated at room temperature. The preserved monodisperse structure indicates the long-term stability of the nanoparticles. The EDX spectrum of CTAB-MTX-OAm-Fe_3_O_4_ nanoparticles obtained from SEM analysis reveals the presence of Fe, O, C, N, and Br elements (Figure 5b–d).

**Figure 6:**
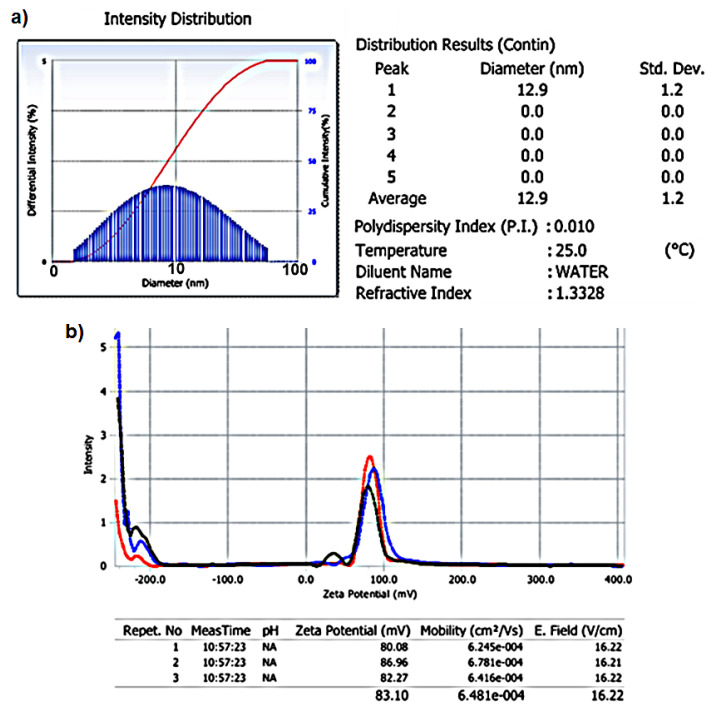
(**a**) DLS result and (**b**) zeta potential of CTAB-MTX-OAm-Fe_3_O_4_ nanoparticles dispersed in water; different colors (red, blue, black) indicate repeated measurements.

The size distribution of CTAB-MTX-OAm-Fe_3_O_4_ nanoparticles was measured as 12.9 ± 1.2 using a zeta-sizer instrument (Figure 6a). This result is higher than the size observed from TEM images (<10 nm) since the instrument measures the hydrodynamic radii [65]. Additionally, according to the TEM images of Fe_3_O_4_ and DLS results of CTAB-MTX-OAm-Fe_3_O_4_ nanoparticles, there was a small increase observed (from 4.7 to 12.9 nm) in mean sizes upon further functionalization.

The zeta potential of CTAB-MTX-OAm-Fe_3_O_4_ nanoparticles in aqueous solution was measured and found to be +83.10 mV (Figure 6b). The result displays the successful functionalization of OAm-Fe_3_O_4_ nanoparticles with a cationic surfactant, CTAB. In addition, the high positive value (+83.10 mV) shows the repulsive power of the nanoparticles and their stability in the aqueous environment since the potential required for the nanoparticle stability to be able to disperse without agglomerating is ±30 mV.

**Figure 7:**
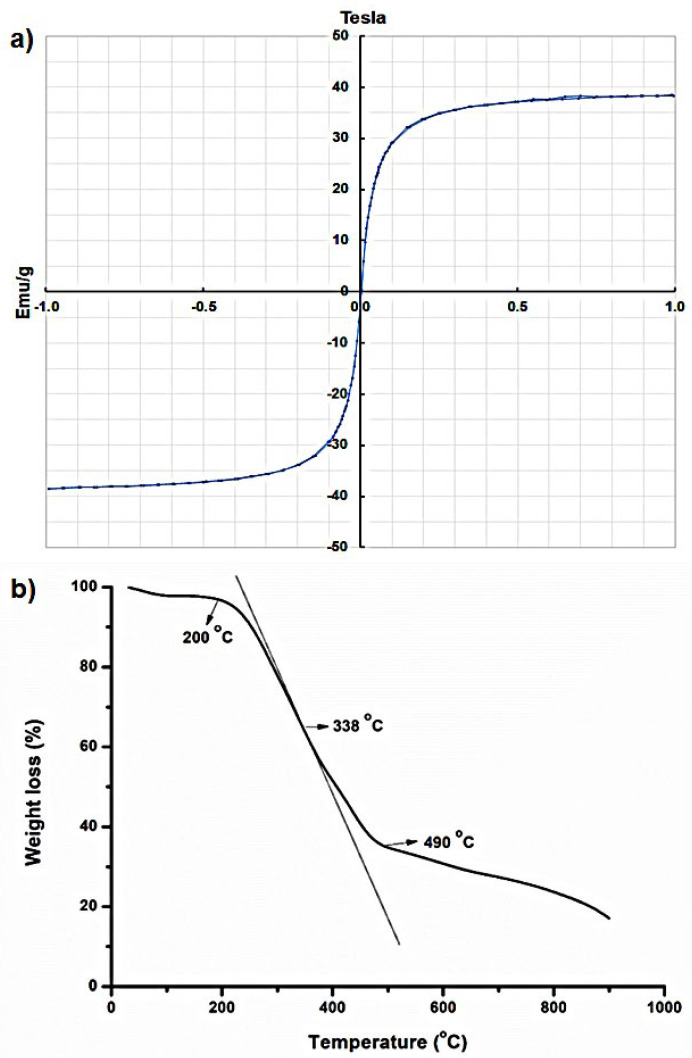
(**a**) Magnetization–hysteresis (M–H) loop and (**b**) TGA curve of CTAB-MTX-OAm-Fe_3_O_4_ nanoparticles.

VSM analysis of the nanoparticle sample was carried out at room temperature and under ±1 Tesla magnetic field intensity (Figure 7a). According to the magnetic hysteresis graph, CTAB-MTX-OAm-Fe_3_O_4_ nanoparticles exhibited superparamagnetic properties, presenting no remanence or coercive forces. The saturation magnetization value (Ms) was found to be 38.4 emu/g from the cycle. According to the literature, Ms value greater than 10 emu/g indicates strong magnetic response and is quite applicable for biomedical studies [66,67]. However, Ms values often range between 30 and 80 emu/g in the literature for some reasons, as well as particle sizes, structure, and morphologies [68]. The alteration in magnetization often stems from the phase conversion of Fe_3_O_4_ that easily oxidizes to a less magnetic phase of Fe_2_O_3_ and the existence of a non-magnetic coating or molecule network over the nanoparticle surface that may cause a magnetically dead area or preserve the superparamagnetic properties [68,69].

Thermogravimetric analysis was carried out to display the stability and ligand functionalization of the CTAB-MTX-OAm-Fe_3_O_4_ nanoparticles (Figure 7b). The first weight loss occurred at 200 °C, which corresponds with the removal of the moisture absorbed by the nanoparticle surface [70]. The significant weight loss of the nanoparticle sample was observed at 338 °C, resulting from the volatilization and combustion of ligands on the nanoparticle surface. An additional weight loss above 490 °C represents the complete degradation of organic compounds [71,72].

**Figure 8:**
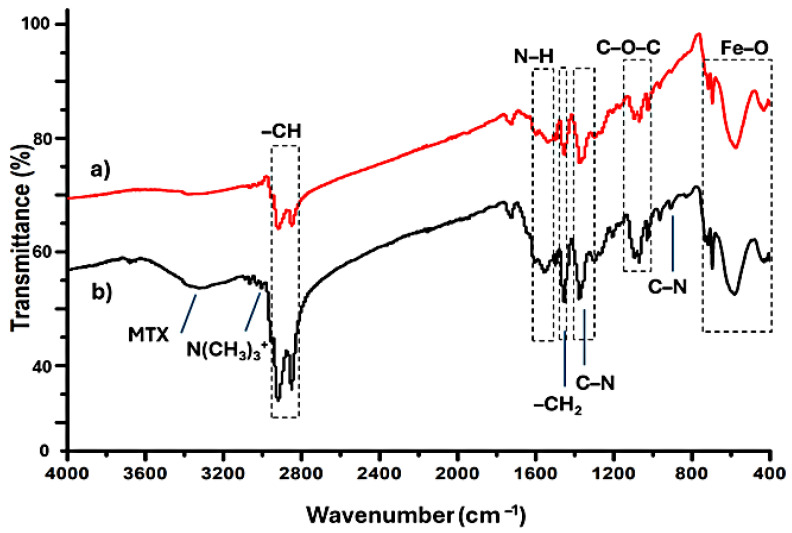
FTIR spectrum of (**a**) OAm-Fe_3_O_4_ (red) and (**b**) CTAB-MTX-OAm-Fe_3_O_4_ nanoparticles (black).

Lastly, FTIR and Raman spectra of OAm-Fe_3_O_4_ and CTAB-MTX-OAm-Fe_3_O_4_ nanoparticles were obtained to elucidate the functional groups on the surface of the nanoparticles. FTIR spectra display the oleyl amine functionalization of Fe_3_O_4_ and further functionalization of OAm-Fe_3_O_4_ using CTAB and MTX (Figure 8). The characteristic absorption peaks of Fe–O at 695, 582, and 430 cm^−1^ were observed for the Fe_3_O_4_ nanoparticle spectrum (Figure 8a) [44,73,74]. After being coated with CTAB, OAm-Fe_3_O_4_ nanoparticles show small shifts in their IR bands (from 695 to 690, 582 to 571, and 430 to 427 cm^−1^), suggesting an interaction between the OAm-Fe_3_O_4_ nanoparticle surface and the CTAB molecules (Figure 8b). The peaks centered at around 1080 cm^−1^ are referred to as C–O–C ether bonds in both spectrums [73]. The peak at 1376 cm^−1^ is attributed to attributed to the C-N bond in both spectrums [75]. The existence of oleylamine in the samples is confirmed by the usual bands of N-H bond vibration at 1600–1500 cm^−1^ seen in all the spectra [73]. The peaks in the 2800–3000 cm^−1^ region is referred to as the –CH_2_ bonds of the saturated alkane in Figure 8a [75]. The intensity increases of the bands at 2876 and 2945 cm^−1^ confirm the CTAB presence in Figure 8b. Asymmetric vibration of –N(CH_3_)_3_ and C-N groups of CTAB emerged at 3017 and 922 cm^−1^ for the CTAB molecule, respectively (Figure 8b) [44]. The additional peak at 3317 cm^−1^ is attributed to the NH group of MTX [74,76,77].

**Figure 9:**
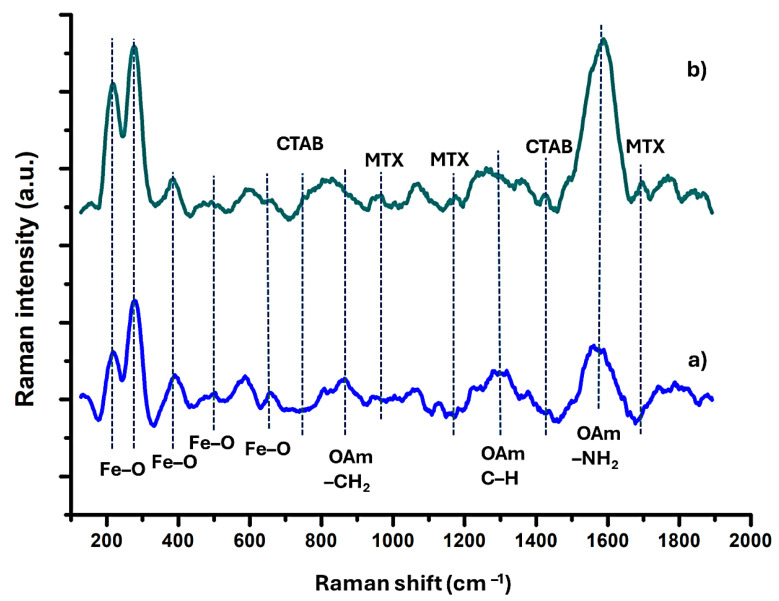
Raman spectrum of (**a**) OAm-Fe_3_O_4_ (blue) and (**b**) CTAB-MTX-OAm-Fe_3_O_4_ nanoparticles (green).

Raman spectra exhibited the signals of magnetite (Fe_3_O_4_), which are dominated by five peaks at around 200 and 650 cm^−1^ [78,79]. Three main peaks at 890, 1300, and 1590 cm^−1^ indicate the OAm presence in the nanoparticle structure in both spectra (Figure 9a,b) [80,81]. The characteristic bands of CTAB disappeared at 763 and 1460 cm^−1^, confirming the presence of the ligand on the surface of the nanoparticle in Figure 9b [82,83]. The additional bands at 961, 1149, and 1700 cm^−1^ in Figure 9b demonstrate the presence of the MTX encapsulated in nanoparticle structure [84,85,86].

### 2.6. Biodegradability of Nanoparticles

**Figure 10:**
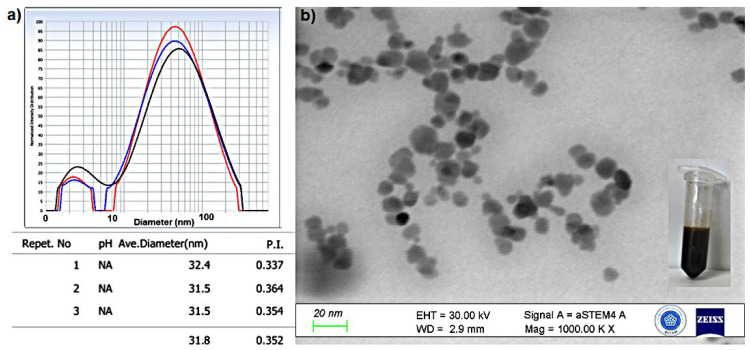
(**a**) DLS result in FBS, PBS (pH 7.4) medium; different colors (red, blue, black) indicate repeated measurements. (**b**) Scanning transmission electron microscope image (STEM), scale: 20 nm, of CTAB-MTX-OAm-Fe_3_O_4_ nanoparticles.

Biodegradability and stability are crucial for storage, usage, and in vivo applications of nanoparticles [87]. The stability of CTAB-MTX-OAm-Fe_3_O_4_ nanoparticles against degradation was assessed in simulated biological media that was 10% FBS in PBS (pH 7.4) at 37 °C by measuring the possible nanoparticle agglomeration and size distribution after 48 h of incubation from DLS measurement and TEM image since the max cellular uptake of nanoparticles has been reached after 48 h. It was observed that CTAB-MTX-OAm-Fe_3_O_4_ nanoparticles were stable and kept their dispersity in the presence of FBS and at 37 °C during 48 h. The size increase and agglomeration were moderate by 48 h, and the mean diameter was determined to be 31.8 nm (Figure 10a,b). This finding is important since the successful delivery of the drug is determined by the nanocarriers’ stability in biological media even after 48 h, which protects the precipitation of nanoparticles during systemic circulation.

### 2.7. In Vitro Experiments

#### 2.7.1. Cytotoxicity Tests

**Figure 11:**
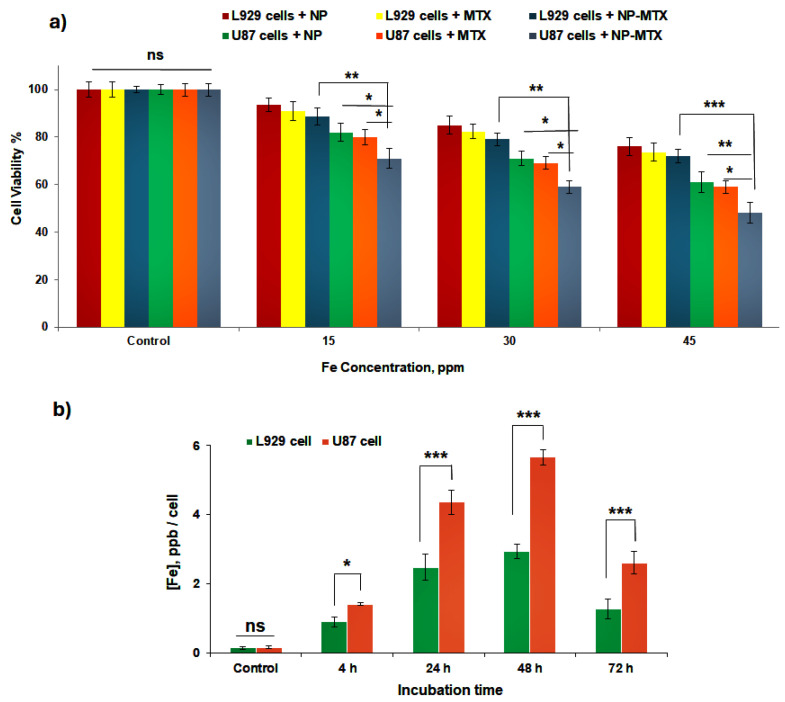
(**a**) Cell viability graph of L929 and U87 cell lines incubated for 24 h with free MTX, CTAB-OAm-Fe_3_O_4_, and CTAB-MTX-OAm-Fe_3_O_4_ nanoparticles at various concentrations (15, 30, and 45 ppm), (CTAB-OAm-Fe_3_O_4_ and CTAB-MTX-OAm-Fe_3_O_4_ nanoparticles are abbreviated as NP and NP-MTX, respectively); (**b**) Graph of ‘Fe concentration per cell (ppb/cell) for U87 and L929 cells incubated with CTAB-MTX-OAm-Fe_3_O_4_ nanoparticles (45 ppm Fe) versus incubation times (4, 24, 48, and 72 h). The data are expressed as mean ± SD and acquired from three biologically independent experiments. * *p* < 0.05, ** *p* < 0.01, and *** *p* < 0.001 compared to untreated cells.

The relative influence of cellular internalization of free MTX, and MTX-loaded (CTAB-MTX-OAm-Fe_3_O_4_) and not-loaded (CTAB-OAm-Fe_3_O_4_) nanoparticles on cell viability of L929 normal and U87 cancer cell lines was studied with the MTT assay. The dose-dependent (15, 30, and 45 ppm Fe) experiments were designed for both cells to reach the half-maximal inhibitory concentration (IC50), and the cytotoxicity profile of cells was presented in Figure 11a. CTAB-MTX-OAm-Fe_3_O_4_ nanoparticles at the concentration of 45 ppm reduced cell viability to 49% in U87 cells and 72% in L929 cells, while this value was found to be 61% in U87 cells and 76% in L929 cells for CTAB-OAm-Fe_3_O_4_ nanoparticles for 24 h. Thus, the ~IC_50_ value was reached for U87 cells incubated with CTAB-MTX-OAm-Fe_3_O_4_ nanoparticles and determined as 45 ppm Fe. As expected, the higher toxicity induced by CTAB-MTX-OAm-Fe_3_O_4_ nanoparticles in U87 cancer cell lines is a promising result for cancer treatment. The IC50 value of 45 ppm (Fe concentration) was utilized for subsequent experiments.

Moreover, the max free MTX concentration was kept identical to the MTX concentration (30.6 µg/mL) loaded into the CTAB-MTX-OAm-Fe_3_O_4_ nanoparticles. Three different concentrations of MTX (30.6, 15.3, and 7.6 µg/mL) displayed a dose-dependent toxicity to both cells (Figure 11a). Free MTX at the concentration of 30.6 µg/mL reduced cell viability to 59% in U87 cells and 74% in L929 cells. MTX-loaded nanoparticles showed a higher inhibitory effect on cancer cells (49%) in comparison with free MTX (59%) and free nanoparticles (61%) (Figure 11a). It is known that MTX is an anti-metabolite agent with a structure very similar to that of folic acid. MTX inhibits the DHFR enzyme, which restricts the synthesis of purines from scratch, DNA replication, and repair. Consequently, these effects of MTX suppress cell division and proliferation [56]. In addition to this, as indicated in Figure 11b, the enhanced cellular uptake of cationic nanocarriers might have increased MTX accumulation and the interaction of MTX and DHFR enzyme after cellular internalization [88]. In addition, Fe_3_O_4_ nanoparticles also have an inhibitory effect with their intrinsic properties, such as the reactive oxygen species (ROS)-generating property, which damages DNA, proteins, and lipids and triggers cell death mechanisms, such as apoptosis or necrosis [89,90]. Cationic Fe_3_O_4_ nanocarriers may disrupt the cell membrane integrity and release iron ions, which promote toxicity and reduce the cell viability [91,92]. All these possible inhibitory substances might create a synergic effect of both cationic Fe_3_O_4_ nanoparticles and the existence of MTX for cellular toxicity.

#### 2.7.2. Cellular Uptake of Nanoparticles

Both cells (U87 and L929) were incubated with CTAB-MTX-OAm-Fe_3_O_4_ nanoparticles at the IC_50_ concentration (45 ppm), and the Fe concentration (ppm) taken up per cell was estimated for 4, 24, 48, and 72 h (Figure 11b). The cellular uptake of nanoparticles started in the first 4 h, and nanoparticles showed a time-dependent cellular uptake in both cell types. The highest amount of uptake was observed at 48 h incubation for both cells. The cellular uptake in U87 is 1.75-fold and 1.92-fold higher than that of L929 at 24 h and 48 h, respectively. The highest uptake in cancer cells is expected since cancer cells have a higher endocytosis potential than normal cells; furthermore, cationic nanoparticles might have the tendency to attach to highly charged (negative) cancerous cell walls. Moreover, the uptake rate gradually slowed down after 24 h for both cells, and a dramatic decrease in the uptake rate was observed after 48 h.

ICP-MS results gave information about the total Fe amount present in the cell medium. However, it does not give information on whether nanoparticles were adsorbed onto the surface of the cell or internalized into the cells [93]. At the initial time points up to 24 h, cellular internalization is higher; however, a kind of saturation of nanoparticles over the cell membrane may occur with time after 48 h upon the limited cellular uptake. Thus, nanoparticles over the cell membrane might have been washed at 72 h. Therefore, the decrease in cellular uptake at 72 h might be due to the absorption of nanoparticles onto the surface of cells. Additionally, membrane integrity may be disrupted in the cancer cells upon both internalization and/or absorption of a high concentration of nanoparticles into the cell or due to cell death, and nanoparticles might be removed from the cell medium upon the washing procedure after 48 h [94]. Moreover, some pathways, including phagocytosis, micropinocytosis, and receptor-mediated endocytosis, such as caveolae-mediated, clathrin-mediated, and caveolae/clathrin-independent endocytosis, may have taken place in cellular uptake mechanisms of nanoparticles [93]. These pathways may be influenced by their cell cycle phases, and these phases are time dependent [95]. Thus, to evaluate these assumptions, microscopic visualization of cells with nanoparticles is needed, and the actual uptake mechanism should be investigated for future studies.

#### 2.7.3. Annexin-V Apoptosis Analysis (Flow Cytometry Analysis)

**Figure 12:**
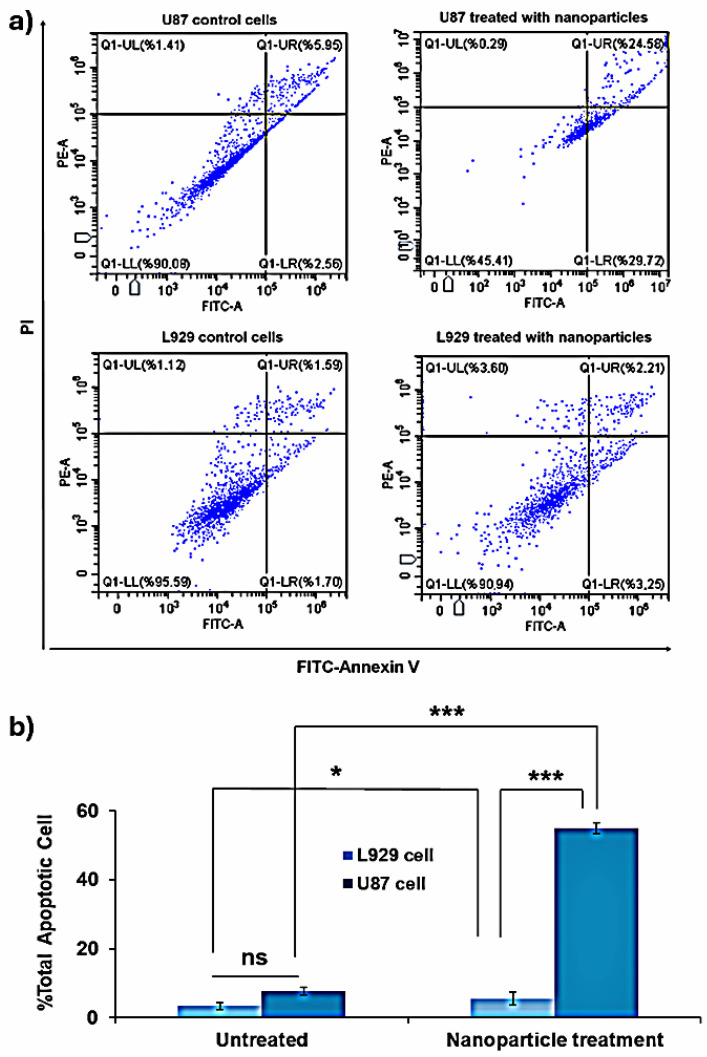
(**a**) Flow cytometry histograms of L929 and U87 cell lines treated with CTAB-MTX-OAm-Fe_3_O_4_ nanoparticles (45 ppm) for 24 h. Untreated cells, used as negative control. The Top left and bottom left reveal necrotic and live cell populations, while top right and bottom right reveal late and early apoptotic cell populations, respectively. Total apoptotic cell populations were estimated from the sum of early and late apoptosis populations, (**b**) Graph showing the percentage of the total apoptotic cell populations for both cell types. The data are expressed as mean ± SD and acquired from three biologically independent experiments. * *p* < 0.05, and *** *p* < 0.001 compared to untreated cells.

Potential cell deaths in L929 and U87 cells treated with CTAB-MTX-OAm-Fe_3_O_4_ nanoparticles (45 ppm Fe) that occurred via apoptotic pathways were investigated by double staining the cells with FITC-Annexin-V and PI dyes. The results of flow cytometry analysis are given as histograms (Figure 12a). The regions in the histogram reveal the distribution of different events in cell lines. A1 and A3 reveal late apoptotic and live cell populations, while areas A2 and A4 reveal dead and early apoptotic cell populations, respectively [94]. Total apoptotic cell populations were obtained by considering both the early and late apoptosis populations from the histograms (Figure 12b). The results show that live cell population is still high (90.94%) for L929 cells while it is decreasing to 45.41% for U87 cells, which was also consistent with the MTT cell viability results for 24 h. The percentage of necrosis did not show a significant difference with an increase of 0.29% for U87cells, while a small increase of 3.60% was observed for L929 cells. The outstanding point of the result is that the total apoptosis percentage in U87 cells is 54.30%, which is ~10-fold higher than that of L929 cells (5.46%). This result indicates the selective apoptosis occurring in U87 cells, which is the expected result of the selective uptake of cationic nanoparticles in cancer cells. CTAB-MTX-OAm-Fe_3_O_4_ nanoparticles have a significant impact in terms of apoptosis at 24 h treatment, whereas the apoptotic rate may increase after 24 h since the cellular uptake of nanoparticles increases up to 48 h to some extent. Therefore, synthesized cationic magnetic nanoparticles can be a promising magnetic delivery agent to the target region at higher concentrations (≥45 ppm Fe) and longer exposure times for selective apoptosis in cancer cells.

#### 2.7.4. Wound Healing Assay

**Figure 13:**
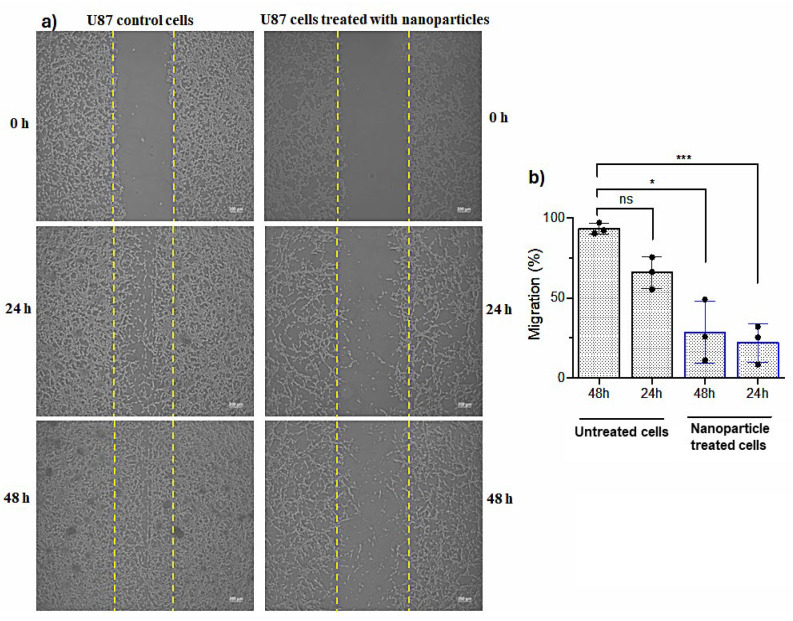
(**a**) Pictures of scratch morphology of the control cells (**left**) and cells treated with CTAB-MTX-OAm-Fe_3_O_4_ nanoparticles (**right**); (**b**) Graph showing the percentage of the migrated cells into the wound area with time. The data in are expressed as mean ± SD and acquired from three biologically independent experiments. * *p* < 0.05, and *** *p* < 0.001 compared to untreated cells.

The wound scratches were created for U87 cells treated with CTAB-MTX-OAm-Fe_3_O_4_ nanoparticles at IC_50_ concentration (45 ppm) and untreated U87 cells. The wells were photographed by light microscope at the beginning, after the nanoparticle treatment of 24th and 48th h (Figure 13a). As expected, cell migration was easily observed in the untreated cells while time-dependent inhibition of the cells was observed by the nanoparticles over time (Figure 13b). Therefore, it can be predicted that CTAB-MTX-OAm-Fe_3_O_4_ nanoparticles inhibited the cell motility and prevented metastasis of U87 cell lines [96].

## 3. Materials and Methods

### 3.1. Materials

Acetylacetonate (Fe(acac)_3_), oleylamine, benzyl ether, hexane, cetyltrimethylammonium bromide (CTAB), methotrexate (MTX), ethanol, 3-(4,5-Dimethylthiazol-2-yl)-2,5-diphenyltetrazolium bromide (MTT), and dimethylsulfoxide (DMSO) were purchased from Sigma-Aldrich (St. Louis, MO, USA). Fetal bovine serum (FBS), trypsin–EDTA, Dulbecco’s Modified Eagle’s Medium (DMEM), penicillin–streptomycin, 1% L-glutamine, non-essential amino acids, and sodium pyruvate were purchased from Gibco (Gibco; Thermo Fisher Scientific, Inc., Waltham, MA, USA). Phosphate buffered saline (PBS) was supplied from Thermo Fisher (Thermo Fisher Scientific, Inc., Waltham, MA, USA) and the annexin V/PI assay kit was supplied by BD (Cat. No. 556547, Franklin Lakes, NJ, USA). Cell lines used in the study, U-87 MG of Glioblastoma Multiforme and L929 as the control, were obtained from the American Type Culture Collection (ATCC).

### 3.2. Synthesis of Iron Oxide Nanoparticles

The thermal decomposition method was used for the synthesis of monodispersed and ultra-small iron oxide nanoparticles in which the chemical reduction of iron (III) (Fe(acac)_3_) takes place at a high temperature in the solution phase in the presence of OAm, which is acting as the reducing agent and surfactant [97]. In the classic procedure, acetylacetonate Fe(acac)_3_ (1 mmol), as a metal source, and OAm (5 mL), as a reducer and surfactant, were used for the synthesis of iron oxide nanoparticles [98]. The synthesis was carried out by dissolving the precursors in an organic solvent with a high boiling point to reach a high decomposition temperature. For this purpose, benzyl ether (5 mL), with a boiling point of 298 °C, was used to reach the decomposition temperature.

All the reagents were mixed under nitrogen gas purge and heated to 100 °C for 1 h to ensure the formation of nanoparticle nuclei. Then, the temperature was increased to 300 °C at a rate of 20 °C/min, and the reaction mixture was kept at this temperature for 1 h. Nucleus growth occurred at this stage. After the synthesis was completed, the suspension was brought to room temperature, and homogeneous-sized nanoparticles were separated from agglomerated particles and excess reducing agent (surfactant) by centrifugation at 9000 rpm and washing with hexane and ethanol. The particles were precipitated with ethanol and dissolved again with hexane and washed 5 times. Lastly, the nanoparticles were suspended in hexane (7 mL).

### 3.3. Phase-Transfer and MTX Incorporation Experiments

Two strategies were designed to incorporate MTX into the nanoparticle surface: The first is the electrostatic adsorption to CTAB-charged tails at the outer surface and physical entrapment of MTX at the interfacial area of oleylamine and CTAB ligands [30,59]. The second way has the possibility of chemical conjugation via hydrophobic interactions of MTX with oleylamine and CTAB besides the physical entrapment [27,56].

CTAB-functionalized OAm-Fe_3_O_4_ nanoparticles were prepared by optimizing the CTAB and nanoparticle concentration, stirring type (mixing or ultrasonic), reaction time, and the order of reactant additions to avoid some agglomerations during the synthesis. The reactions were carried out by keeping all the parameters constant except one to be investigated. It was preferred to reach maximum CTAB concentration for the maximum drug encapsulation due to some possible dissociation of the ligand in time. In the optimized reaction, 1 mL of nanoparticle suspension was obtained by the thermal decomposition method, and 1 mL of CTAB at a concentration of 50 mg/4 mL was used. The hexane suspension of the iron oxide nanoparticles (750 µL) was evaporated with N_2_ gas and taken into 1000 µL chloroform and kept in an ultrasonic bath for 30 min. CTAB solution (1 mL) was added to 1 mL of nanoparticle suspension and kept in an ultrasonic bath for 3 h. After the reaction was completed, the nanoparticles were washed with hexane by centrifugation at 9000 rpm for 10 min. The cloudy supernatants, including excess/dissociated CTAB observed in the hexane phase, were discarded. The washing process was repeated 5 times with hexane until the supernatant became transparent, and then the supernatant was discarded. The pellet was dissolved in deionized water, named CTAB-OAm-Fe_3_O_4_ nanoparticles.

In a drug adsorption experiment, 1 mL of CTAB-OAm-Fe_3_O_4_ nanoparticles and the aqueous solution of MTX at the concentration of 1 mg/mL were mixed on a magnetic stirrer for 1 h at room temperature to ensure the attachment of drug molecules to the nanoparticle surface via electrostatic interaction. Here and in the entrapment experiments below, MTX amount with various volumes ranging between 10 and 100 µL was optimized for a maximum drug loading amount. After the reaction was completed, the nanoparticles were washed with ethanol three times by centrifugation at 9000 rpm for 10 min. The pellet was then dissolved in deionized water.

In an entrapment experiment, the optimized concentrations of reactants (OAm-Fe_3_O_4_ and CTAB) above were utilized. The Fe_3_O_4_ nanoparticles were transferred to 1000 µL of chloroform and kept in an ultrasonic bath for 30 min. The aqueous solutions of MTX (with various volumes ranging between 10 and 100 µL) at the concentration of 1 mg/mL and 1 mL of CTAB at the concentration of 50 mg/4 mL were added to 1 mL of OAm-Fe_3_O_4_ nanoparticle suspension simultaneously. The mixture was kept in an ultrasonic bath for 3 h. After the reaction was completed, the nanoparticles were washed with hexane three times by centrifugation at 9000 rpm for 10 min. The pellet was then dissolved in 2 mL of deionized water.

The quantification of MTX incorporated into the nanoparticles obtained by both methods (physical entrapment and surface adsorption) was indirectly evaluated by keeping the MTX amount constant (50 µL, 1 mg/mL) in the synthesis, and then MTX-incorporated nanoparticles were centrifuged at 30,000 rpm for 30 min using an ultracentrifuge. MTX in supernatant was analyzed using a UV–Vis spectrophotometer at its specific wavelength of 305 nm. Incorporated MTX was calculated by considering the concentration of MTX initially added to the nanoparticle suspension and in the free MTX in the supernatant. The yield percentage of MTX for both methods is calculated from the following equation [32]:

Yield % = [(CMTX (initial) − CMTX (free))/(CMTX (free)] × 100

MTX-incorporated CTAB-OAm-Fe_3_O_4_ nanoparticles are called CTAB-MTX-OAm-Fe_3_O_4_.

### 3.4. Determination of Incorporation Efficiency

The HPLC method was used to determine the incorporation efficiency of MTX for nanoparticles synthesized by the entrapment experiment since the drug loading amount was found to be higher than that of the physical adsorption way. The samples prepared with a range of MTX content (10, 25, 50, 75, 100 µL) were centrifuged at 30,000 rpm for 30 min using an ultracentrifuge and dialyzed against PBS buffer (pH: 7.4) for 6 h. The samples were analyzed for their MTX content by the HPLC method. Before loading the samples to the HPLC column, CTAB-MTX-OAm-Fe_3_O_4_ nanoparticle samples were dissolved in HCl to release the drug molecules and then diluted 10-fold with deionized water. The solution was filtered with a 0.44 µm filter syringe to remove large particles.

Studies were carried out on a Shimadzu LC-10Atvp system equipped with a Thermo ODS-2 HYPERSIL (250 × 4.6) column and an SPD-M20A diode array detector. A 0.1% TFA-water (solvent A) and methanol (solvent B) (66:34, *v*/*v*) were used as mobile phases. The UV detector was operated at a wavelength of 305 nm. The temperature was applied as 25 °C and the flow rate was 1 mL/min. MTX standards were prepared at five different concentrations, including 31.25 µg/mL, 62.5 µg/mL, 125 µg/mL, 250 µg/mL, and 500 µg/mL. The areas under the MTX peaks in the chromatograms obtained for each standard were measured, and a calibration curve was created [99].

The drug incorporation efficiency of CTAB-MTX-OAm-Fe_3_O_4_ nanoparticles was calculated according to the following formula [32]:

IE % = ((C_MTX_ (initial) − C_MTX_ (free))/(C_MTX_ (initial)) × 100

IE %: Percentage of drug incorporated within the nanoparticle structure.

C_MTX_ (initial): Amount of drug loaded into the nanoparticle suspension.

C_MTX_ (free): Amount of drug remaining in the supernatant solution after centrifugation and a 6 h dialysis.

### 3.5. Drug Release Studies

Experiments were designed from literature with some modifications [100,101,102]. The drug release profile was evaluated for CTAB-MTX-OAm-Fe_3_O_4_ nanoparticles prepared with the predetermined maximum amount of MTX (75 µL, 1 mg/mL) incorporated. After removal of the free MTX by centrifugation, samples were dissolved in deionized water, and 1 mL of sample was placed in a dialysis membrane (MWCO: 12 kDa). Samples were dialyzed against 5 mL of PBS buffer (pH 7.4) or acetate buffer solution (pH 5.0) for 72 h. For the analysis of the amount of MTX released, 500 µL of the released medium was collected at time intervals of 0, 3, 6, 12, 24, 48, and 72 h for HPLC analysis, and then the external buffer was returned to its initial volume to maintain a constant volume. The released drug concentration was calculated using the HPLC standard calibration curve experimentally obtained. Results are given as averaged values from three independent experiments. MTX release % is calculated as the following equation [102]:

MTX release % = ((C_MTX_ (released)/(C_MTX_ (initial)) × 100

### 3.6. Stability Test in Human Serum

A total of 500 mL of CTAB-MTX-OAm-Fe_3_O_4_ nanoparticle suspension was combined with 500 mL of PBS (pH 7.4) containing FBS (10%, *v*/*v*) and incubated in a shaker incubator at 37 °C for 1 h to assess the stability and degradability of nanoparticles in a simulated physiological media. Nanoparticles were then left at room temperature and at 48 h post incubation, the nanoparticle suspension was used for DLS measurement and TEM imaging [103,104].

### 3.7. Characterization Experiments

High-contrast transmission electron microscopy (CTEM, FEI Tecnai G2 Spirit Biotwin, FEI, Hillsboro, OR, USA) with an accelerating voltage of 120 kV was used for shape and size analysis of OAm-Fe_3_O_4_ nanoparticles by dropping 10 µL of the nanoparticle suspension in hexane onto a carbon-coated copper grid. Elemental composition of nanoparticles was also elucidated by an energy-dispersive X-ray spectroscopy (EDS) analysis with a field emission scanning electron microscope (FESEM, ZEISS Gemini SEM 500, Carl Zeiss, Jena, Germany) with an accelerating voltage of 10 kV. Prior to analysis, 10–20 µL of the samples were coated with a 4.30 nm thick layer of iridium on a carbon band. Scanning transmission electron microscopy (STEM, ZEISS Gemini SEM 500) was used for size and shape analysis of the CTAB-MTX-OAm-Fe_3_O_4_ nanoparticles suspended in FBS medium. The specific absorbance band of Fe_3_O_4_ nanoparticles was displayed using a UV–Vis spectrophotometer (Agilent Technologies Cary 60, Santa Clara, CA, USA). The crystal structure of nanoparticles was elucidated by an X-ray diffraction instrument (XRD, PANalytical EMPYREAN) with Cu Ka radiation (40 kV and 30 mA) for 2θ values over the range of 20–70. Surface charges of nanoparticles were measured by a zeta potential analyzer (Micromeritics-Nanoplus 3) at 25 °C. The thermal stability of nanoparticles was investigated by thermogravimetric analysis-differential scanning calorimetry (TGA-DSC, Seteram-Labsys Evo) under oxygen at the temperature range of 25–900 °C with the heating rate of 10 °C/min. The functional groups of the nanoparticles were elucidated using FTIR (Thermo Scientific—Nicolet iS20) and Raman (Renishaw inVia Reflex Confocal, Renishaw, Hong Kong, China) spectrometers. After dissolving nanoparticle suspension in concentrated HCl overnight, quantitative analysis of iron (Fe) in the suspension was determined by inductively coupled plasma mass spectrometry (ICP-MS, Agilent Technologies 7900).

### 3.8. In Vitro Studies

#### 3.8.1. Cell Culture

U-87 MG of glioblastoma multiforme and L929 (fibroblast) cell lines as a control were cultured in DMEM (Dulbecco’s Modified Eagle Medium) supplemented with 1% L-glutamine, non-essential amino acids, sodium pyruvate, 10% fetal bovine, and 1% PSA (penicillin/streptomycin/amphotericin) in a 5% CO_2_ atmosphere, 95% humidity, and 37 °C incubator.

#### 3.8.2. In Vitro Cytotoxicity

The cytotoxic effects of CTAB-MTX-OAm-Fe_3_O_4_ nanoparticles on L929 and U87 cells were determined by MTT (3-(4,5-dimethylthiazol-2-yl)-2,5-diphenyltetrazolium bromide) assay [94,105]. For this purpose, cells were seeded in 96-well culture dishes at 105 cells/well and cultured overnight. The adherent cells in the wells were treated with different concentrations (22.5, 45, and 67.5 ppm) of CTAB-MTX-OAm-Fe_3_O_4_ nanoparticles and incubated for 24 h. After the nanoparticle incubation, 10 μL/well of MTT solution (5 mg/mL) was added to the cells and incubated for an additional 3 h. Then, the culture medium was removed from the cells, and 100 μL of dimethyl sulfoxide (DMSO) was added to each well to dissolve the MTT salt. After 15 min incubation, the optical density (OD) of MTT at 570 nm was measured using an ELISA reader based on the color change resulting from the reduction of MTT salt by viable cells. These procedures were conducted for each cell line with three replicates. Untreated cells with nanoparticles were used as the control. Using OD values, the viability levels of the cells exposed to different concentrations of nanoparticles were determined, and the IC_50_ concentration value of the nanoparticle suspension was calculated. The cytotoxic effect of nanoparticles on cells was evaluated from cell viability percentage, which is determined by the following formula [106]:

Cell Viability Percentage (%) = (Average OD/Control OD) × 100

where

Average OD: Average absorbance value of the nanoparticle-treated cells;

Control OD: Absorbance value of the untreated control cells.

#### 3.8.3. Cellular Internalization (Inductively Coupled Plasma Mass Spectrometry)

For quantitative analysis of cellular internalization of nanoparticles, cells (2 × 10^6^) in 96-well culture dishes were incubated with nanoparticles at the IC_50_ concentration (45 ppm Fe) for 4, 12, 24, and 48 h in separate experiments. Then cells were washed three times with phosphate buffer (PBS, pH: 7.4) and trypsinized, stained with trypan blue, and counted under light microscopy. All the cells treated in the same way were digested with 1 mL of HCl overnight and then diluted with deionized water for Fe analysis by ICP-MS (Agilent Technologies 7900) instrument. Results were calculated as [Fe], ppb/cell number [94,105].

#### 3.8.4. Annexin-V/PI Staining (Flow Cytometry Analysis)

The instructions of the commercial FITC-Annexin V apoptosis kit with PI (Biolegend, San Diego, CA, USA) were used to evaluate the apoptotic effects of CTAB-MTX-OAm-Fe_3_O_4_ nanoparticles on L929 and U87 cells [94,105]. The cells (1 × 10^6^) were treated with nanoparticles at the IC_50_ concentration (45 ppm Fe) for 24 h. Then, trypsinized cells were suspended, washed with PBS, and resuspended with binding buffer (140 mM NaCl and 2.5 mM CaCl_2_ in 10 mM HEPES/NaOH, pH 7.5). Fluorescein isothiocyanate (FITC)-conjugated Annexin V/propidium iodide (PI) were added to the cell suspensions, and the cells were stained. Stained cells undergoing each event (early apoptotic, necrotic phase, or late apoptotic) were determined by using a BD C6 flow cytometer (Becton-Dickinson, Franklin Lakes, NJ, USA). The control group was composed of cells that were not treated with nanoparticles (untreated). The results are given as histograms.

#### 3.8.5. Migration Experiment

U-87 cells were seeded in 6-well plates at a density of 1 × 10^6^ cells to be confluent. After 24 h of incubation, the wound area was generated with the help of an automatic pipette tip by a linear scratch in the cells. The cells were then washed with 1× PBS three times and treated with CTAB-MTX-OAm-Fe_3_O_4_ nanoparticles at the IC_50_ concentration, kept at 37 °C with 5% CO_2_. The cells without nanoparticles served as a control experiment (untreated). The microscope images of treated and untreated cells were taken using an inverted microscope (Olympus CK40) on day 0, after 24 h, and after 48 h. The distance between adjacent cell layers was evaluated, and the wound areas were measured using Image-J analysis software (version 1.47, NIH); the wound closure percentage was calculated using the following formula [107]:

Wound Closure (%) = (A_0_ − A_t_)/A_0_ × 100,

where

A_0_: the scratch area recorded at 0 h;

A_t_: the scratch area recorded at time ‘t’.

#### 3.8.6. Statistical Analysis

For statistical analysis, GraphPad Prism VI statistical software (GraphPad Inc., La Jolla, CA, USA) was used by using one-way analysis of variance (ANOVA). A *p*-value of less than 0.05 (*p* < 0.05) was regarded as statistically significant. The assays were performed in triplicate, and the results were interpreted as mean ± standard error.

## 4. Conclusions

To the best of my knowledge, this is the first study of its kind to synthesize a simple, effective method with an enhanced total MTX loading by two-way incorporation and a one-step process reported for the surface functionalization, phase transfer, and drug incorporation of nanoparticles, simultaneously. It would be a prototype for other hydrophobic drugs or active molecules, and the findings obtained would lead to new drug encapsulation strategies.

Here, magnetic OAm-Fe_3_O_4_ nanoparticles in hexane were successfully transferred to the water phase without aggregation by ligand functionalization with CTAB, while entrapping MTX at the OAm/CTAB interface and electrostatically attaching on the surface CTAB cationic tails occurred at the same time. From the spectroscopic characterizations of nanoparticles, it has been shown that the nanoparticle maintains its monodispersity and stability with an effective amount of drug loading. In addition, a maximum incorporation efficiency% of 89.5% is found for 25 µg/mL of MTX loaded into nanoparticle suspension. The cumulative amount of drug release reached 40% up to 12 h at physiological pH and 85% up to 12 h at a pH of 5.0 with a significant amount of enhanced drug release. This can reduce the side effects on normal cells since MTX will be readily released in the tumor environment at a pH of 5.0. According to the in vitro experiments, results indicate that CTAB-MTX-OAm-Fe_3_O_4_ nanoparticles decreased cell viability to 49% in U87 cell lines and 72% in L929 cell lines. It is evident that the charged surface is contributing to the cellular internalization of nanoparticles; it is 1.92-fold higher in U87 than that of L929 cells. Nanoparticles are also inducing cell death through apoptosis. Importantly, the total apoptosis percentage in U87 cells is 54.30%, which is ~10-fold higher than that of L929 cells (5.46%). Thus, nanoparticles cause selective apoptosis and necrosis in cancer cells at IC50 concentration, and this feature can also be used in cancer treatment. Lastly, the nanoparticles inhibited the cell motility and prevented metastasis of U87 cell lines via a wound healing assay. In conclusion, the promising in vitro results combined with the stable structure and effective drug release behavior make CTAB-MTX-OAm-Fe_3_O_4_ nanoparticles valuable theranostic candidates.

## Data Availability

The original contributions presented in the study are included in the article; further inquiries can be directed to the corresponding author.
